# Advances in Computational Intelligence Techniques-Based Multi-Intersection Querying Theory for Efficient QoS in the Next Generation Internet of Things

**DOI:** 10.1155/2023/1388425

**Published:** 2023-07-08

**Authors:** Ashish Kumar, Kannan K, Mamta Dahiya, Virendra Singh Kushwah, Ayesha Siddiqa, Kiranjeet Kaur, Saima Ahmed Rahin

**Affiliations:** ^1^Department of CSE, Manipal University Jaipur, Jaipur, Rajasthan, India; ^2^Department of CSE, Sree Vidyanikethan Engineering College, Tirupati, Andhra Pradesh, India; ^3^Department of Computer Science and Engineering, SGT University, Gurugram, India; ^4^School of Computing Science and Engineering (SCSE), VIT Bhopal University, Sehore, India; ^5^Artificial Intelligence and Data Science Department, Islamia Engineering College, Hyderabad, Telangana, India; ^6^Department of CSE, University Centre for Research & Development, Chandigarh University, Mohali, Punjab 140413, India; ^7^United International University, Dhaka, Bangladesh

## Abstract

An environment of physically linked, technologically networked things that can be found online is known as the “Internet of Things.” With the use of various devices connected to a network that allows data transfer between these devices, this includes the creation of intelligent communications and computational environments, such as intelligent homes, smart transportation systems, and intelligent FinTech. A variety of learning and optimization methods form the foundation of computational intelligence. Therefore, including new learning techniques such as opposition-based learning, optimization strategies, and reinforcement learning is the key growing trend for the next generation of IoT applications. In this study, a collaborative control system based on multiagent reinforcement learning with intelligent sensors for variable-guidance sections at various junctions is proposed. In the future generation of Internet of Things (IoT) applications, this study provides a multi-intersection variable steering lane-appropriate control approach that uses intelligent sensors to reduce traffic congestion at many junctions. Since the multi-intersection scene's complicated traffic flow cannot be accommodated by the conventional variable steering lane management approach. The priority experience replay algorithm is also included to improve the efficiency of the transition sequence's use in the experience replay pool and speed up the algorithm's convergence for effective quality of service in the upcoming IoT applications. The experimental investigation demonstrates that the multi-intersection variable steering lane with intelligent sensors is an appropriate control mechanism, successfully reducing queue length and delay time. The effectiveness of waiting times and other indicators is superior to that of other control methods, which efficiently coordinate the strategy switching of variable steerable lanes and enhance the traffic capacity of the road network under multiple intersections for effective quality of service in the upcoming IoT applications.

## 1. Introduction

With the continuous increase in the number of motor vehicles in my country, the contradiction between the supply and demand of road traffic is increasingly intensifying. Especially in the intersection scene, the traffic flow of each turn at the intersection presents an uneven distribution at different time periods, which easily leads to congestion and waste of lanes resources. In order to solve this problem, the variable steerable lane technology using intelligent sensors came into being, which uses the lane as a variable space resource and dynamically allocates it according to the needs of each turning traffic flow on the basis of communications between the wireless sensors, so as to improve the utilization rate of road space resources. An intelligent sensor is a function that can sense and detect information from a specific item, as well as learn, judge, and receive signals, and has a new type of sensor with management and communication features. The intelligent sensor is capable of autonomously calibrating, compensating, and collecting data. The intelligent sensor's capabilities determine its high precision and resolution, stability and dependability, and flexibility. It provides a great performance-to-price ratio when compared to standard sensors. Smart sensors come in three varieties: those that can judge; those that can learn; and those that can be creative. Intelligent velocity sensors, intelligent acceleration sensors, intelligent flow sensors, intelligent position sensors, intelligent attitude sensors, intelligent displacement sensors, and intelligent dimension sensors are a few examples of intelligent sensor types.

Variable steerable lanes at a single intersection of the scene, the traditional control method can effectively alleviate the problem of unbalanced steering, but as the number of variable steering lanes increases, the traffic flow changes between multiple intersections become more complex, and the present ability of the traditional method is more difficult, and it is impossible to coordinately control multiple. Therefore, how to make the cooperation between the multi-intersection variable-guidance lanes more efficient becomes a new problem. Variable steering lane with many intersections is a technique for figuring out the length of a changeable guide lane for a signal control junction approach is disclosed in an invention in the field of road traffic sign marked lines. The process entails first obtaining traffic data information on the variable guide lane's steering in the desired direction, followed by translating the received traffic volume into an equal standard small vehicle traffic volume. Using a model in the theory of queuing, it is then possible to determine the number of vehicles queuing behind a stop line for the signal control intersection approach using a multipath queuing multichannel system and a combination of an approach lane queuing imbalance coefficient. Finally, the vehicle number is converted to a measurement of the span of the variable guide lane for the signal control intersection approach using the vehicle average queuing length in meters. The approach traffic efficiency may be increased by using the technique for estimating the length of the variable guide lane for the signal control intersection approach, which allows cars on the upper stream of the variable guide lane to enter the intersection approach smoothly.

The term “variable-guided vehicle route” describes a track where the travel direction is not totally set for left-hand rotation, right turns, or continuing straight ahead, but instead incorporates various turning functions depending on the time of day. The overall traffic efficiency in the crossing can be greatly increased thanks to the way this track is laid out. Since the entrance driveway to the signalized crossing is not suitable for widening or having many canalizations, the wagon flow lacks uniformity significantly and is not suitable for the condition under which a signal control device can solve the problem. Apply in places like Hangzhou, Wuxi, Tangshan, and Yantai, China's equivalent of Shanghai. Variable lanes are now being installed at crossroads in several places in order to better use road resources and increase traffic capacity. The variable lane control method primarily employs manual observation of traffic conditions for switching, or switching is performed at a fixed-time based on statistics of historical data. The manual switching mode is inefficient, and the statistical technique does not accurately reflect current traffic conditions. However, the rapid development of artificial intelligence and vehicle-road cooperation technologies can supply more real-time and precise data for variable lane control. It is feasible to regulate the variable lane more efficiently by making proper use of these data.

In this paper, we present a collaborative control system for variable-guidance sections at various intersections based on multiagent reinforcement learning and intelligent sensors. This study proposes a multi-intersection variable steering lane-appropriate control strategy that leverages intelligent sensors to minimize traffic congestion at multiple junctions in the next generation of Internet of Things (IoT) applications. The priority experience replay algorithm is also incorporated to increase the efficiency of the transition sequence's utilization in the experience replay pool and accelerate the algorithm's convergence for effective quality of service in forthcoming IoT applications.

## 2. Related Work

The traffic flow at the intersection changes dynamically in time and space. For example, in the morning and evening rush hours, the traffic flow of each turn at the intersection shows obvious regular changes, and there is a serious imbalance in the queue length of vehicles in different guide lanes. In order to improve the traffic capacity of the intersection and solve the problem of urban road congestion, some traffic researchers have carried out research on the dynamic control method of variable steering lane steering, mainly focusing on three aspects: traditional control method, intelligent control method, and reinforcement learning method. Supervised learning is a machine learning term that refers to the approach of constructing a function independently by learning from a number of related samples. This is the method of learning a broad notion from a small number of examples that are comparable to others. Contrarily, reinforcement learning is a branch of machine learning that builds on the idea of behavioral psychology and focuses on interacting with the environment directly. It is an important part of the field of artificial intelligence. Regression and classification are the two major jobs in supervised learning, whereas exploitation or exploration, Markov's decision processes, policy learning, deep learning, and value learning are the diverse tasks in reinforcement learning. Basic reinforcement is specified in the model Markov's decision process in reinforcement learning, whereas supervised learning examines the training data and generates a generalized formula. Each example in supervised learning will have a pair of input objects and an output with the desired values, whereas, in reinforcement learning, the agent interacts with the environment in discrete steps, making an observation for each time period “*t*,” receiving a reward for each observation, and then attempting to accumulate as many rewards as possible in order to make more observations.

The formula to measure the queuing length of the lanes can be explained as we can demonstrate for the M/M/1 queue that *L*_*q*_=*ρ*2/1 − *ρ*

We can demonstrate for the M/G/1 queue that

(1) $$\begin{array}{c} L_{q}=\frac{\lambda^{2}\sigma_{s}^{2}+\rho^{2}}{2\left(1-\rho \right)}\text{,} \end{array}$$

where *L*_*q*_ is the mean number of customers in the queue, the likelihood that it is busy, and the percentage of time it is busy are all represented by the formula *ρ* = *λ*/(*cµ*), 1/*E* [Inter-arrival-Time], where *E* stands for the expectation operator, represents the mean rate of arrival, and *σ*_*s*_^2^ variance of service time.

### 2.1. Traditional Control Method

The traditional variable lane control method research uses the method based on experience or historical data to set the control plan in advance and design the rules for the steering of the variable steerable lane at the intersection. Literature [1] proposed a signal for lane optimization at the intersection based on experience with phase-integrated design. Literature [2, 3] proposed a steering control model for a variable-steering lane at an intersection based on the empirical rules for the setting conditions of the variable-steering lane. The change characteristics of traffic flow and the actual traffic steering demand are more closely matched in Literature [4] and comprehensively considered the real-time traffic factors at a single intersection, and evaluated the preimplementation plan.

The specific lane function and signal timing switching scheme are deployed, but the preplan must be repeatedly tested and the accuracy is not high. Literature [5, 6] carried out integer nonlinear programming according to multiple road constraints associated with the target intersection. The optimization of the model achieves the smallest critical flow ratio after the optimization of a single intersection. Literature [7, 8] integrated the road conditions of key intersections and downstream adjacent intersections to achieve an associated control model. The above-mentioned work only considers the key intersection to a single adjacent intersection impact but does not design a comprehensive optimization scheme for associated intersections. Literature [9] proposed a control method to coordinate the design of multiple intersection variable signs and corresponding signal groups based on the collected data rules to better reduce the average delay of vehicles.

The abovementioned method of presetting the steering rules for variable steering lanes through experience or summarizing historical data rules can adapt to the needs of regular traffic state changes to a certain extent, but it is difficult to dynamically adapt to road traffic conditions and sudden changes in supply and demand abnormal traffic flow.

### 2.2. Intelligent Control Method

Research on intelligent control methods for variable steering lanes makes intelligent decisions based on various traffic flow data collected in real-time, and improves the adaptability to real-time traffic flow changes at intersections. Some works use the collected real-time traffic flow data on roads, such as the space of each turning lane, occupancy rate [10], traffic flow, speed, queue length, and other characteristics obtained through video detection [11], dynamic decision-making variable steering lane switching strategy, but its adaptability to subsequent traffic flow changes is not good when combined with real-time collected data. Literature [12] predicted each turning traffic flow as the basis for judging lane direction switching and minimized the average delay time at the intersection. Literature [13] used the least squares dynamic weighting and the short-term traffic flow prediction model with fusion algorithm as the core combined with the traffic state prediction model with fuzzy data theory and neural network system theory as the core to realize automatic control of variable steering lane steering. Literature [14] constructed a mixed integer and the two-layer programming model is solved by a particle swarm algorithm to achieve the goal of minimizing the total travel time of variable lanes based on the prediction model.

The above-related research work has two limitations: (1) it is mainly applied to intelligent control decision-making of variable-steering lane steering at a single intersection and (2) the prediction-based intelligent algorithm is mainly based on historical and real-time data, and cannot quickly update rules to adapt to the dynamics of traffic flow variety.

### 2.3. Reinforcement Learning Methods

In recent years, reinforcement learning technology has developed rapidly. It has low requirements for prior knowledge of the environment and can achieve good learning optimization performance in complex nonlinear systems. Therefore, it is suitable for complex and changeable multi-intersection and variable-guided lane intelligent control scenarios. In the multi-intersection collaborative control problem, traffic signal optimization research has widely used the reinforcement learning method. Literature [15] combined deep reinforcement learning with the traffic signal control problem, respectively, defining the state, action space, and reward function, using DQN (Deep Q-Network) model, extensive experiments on synthetic and real datasets demonstrate the superiority of reinforcement learning methods. Literature [16] used multiagent reinforcement learning technology to define the joint *Q*-value as the weighted sum of local *Q*-values, by minimizing the weighted sum of individual *Q*-values and the global *Q*-value to ensure that a single agent can take into account the learning process of other agents and realize automatic control of large-scale traffic signals. Literature [17] proposed that different agents exchange strategies after each round of learning to achieve a zero-sum game. Based on this realize the signal control strategy of autonomous vehicles, and designed a rewarding method that combines individual efficiency with overall efficiency. In the multi-intersection collaborative control scenario, the traffic signal optimizes the traffic situation in the time dimension, and the intelligent variable guide lane is used as the spatial dimension. The above-mentioned two directions are suitable for the use of reinforcement learning methods to carry out global optimization research.

## 3. Variable Steering Lane Cooperative Control Method Employing Intelligent Sensors

### 3.1. Overall Structure

This research proposes a multi-intersection variable-direction lane cooperative control algorithm using intelligent sensors based on multiagent reinforcement learning. The method mainly includes multiagent reinforcement learning model merged with intelligent sensors, a global reward decomposition algorithm, and a priority experience playback algorithm. The multiagent reinforcement learning model proposed is constructed based on the value function decomposition algorithm of the QMIX algorithm [18]. The QMIX algorithm adopts the strategy of centralized training and distributed execution and uses the global reward function to optimize joint actions during training with the aid of intelligent sensors. The value function can achieve the effect of multiagent cooperative control, and each agent constructs and extracts the corresponding local strategy from the joint action-value function, which can not only deal with the problems caused by environmental nonstationary through centralized training but also through joint action-value function backpropagation learns the local “best” policy for each agent, enabling multiagent decentralized execution. Through the provision of a more flexible version of the constraint, QMIX enhances the VDN algorithm. The constraint is described as follows:

(2) $$\begin{array}{c} \frac{\partial \;Qtot}{\partial \;Qa}\geq \text{,} \end{array}$$

where *Q*_tot_ denotes the total value function and *Q*_*a*_ denotes the value function for each agent. The weights of each particular value function *Q*_*a*_ should be positive, according to an obvious explanation. If the weights of the individual value function *Q*_*a*_ are negative, the agent will be discouraged from cooperating since the greater *Q*_*a*_, the lower the joint value *Q*_tot_. For consistency between the centralized and decentralized rules, QMIX comprises agent networks that represent each *Q*_*a*_ and a mixing network that brings them together into *Q*_tot_ rather than just adding them up like in VDN. By requiring the mixed network to have positive weights, it also imposes constraints. Since QMIX's factored representation grows well with the number of agents, it is able to describe complicated centralized action-value functions and makes it simple to extract decentralized policies using individual argmax operations in linear time.

The global reward decomposition algorithm improves the global reward distribution method in the value function decomposition algorithm and imposes constraints between the global value function and the value function of a single agent. In some complex scenarios, the global optimal joint action may require the intelligent sensor to make some behaviors that sacrifice individual interests. Decomposition techniques change a difficult problem into a simpler one. Only binary constraints, whose scopes form a directed acyclic graph, are present in the new problem. Each set of variables from the original problem is represented by a variable in the new problem. These sets encompass the set of the initial variables even if they are not necessarily disjoint. In relation to each set of variables, the translation uncovers all partial solutions. The interplay between local solutions is reflected in the translation problem. A decomposition approach creates a binary acyclic issue by definition; these problems may be solved in a time that is polynomial in their size. In response to this problem, this study decomposes the global reward into two parts, one part is the basic reward, and the specific distribution to different agents is realized through the QMIX hybrid network; the other part is the performance reward, according to the agent. The state hierarchically is assigned to each agent which is the IoT so that a single agent can maximize the global reward while taking into account its own reward, and realize the secondary distribution of the global reward. In RL, the agent receives a reward that is often a sum of many reward components, each designed to encode some aspect of the desired agent behavior. From this composite reward, it learns a single composite value function. Using value decomposition, an agent learns a component value function for each reward component. To perform policy optimization, the composite value function is recovered by taking a weighted sum of the component value functions. While prior work has proposed value decomposition methods for discrete-action *Q*-learning. The development of autonomous agents is frequently done via reinforcement learning. In the RL framework, the agent is permitted to behave in the environment and is rewarded numerically at each step rather than being explicitly programmed. The goal of RL algorithms is to discover a strategy that maximizes the overall predicted reward (or some related criterion). Therefore, the reward function implies that optimal behavior is described. In order to assess actions in terms of trade-offs among the kinds, the technique decomposes rewards into sums of semantically relevant reward categories. To concisely describe why one action is preferable over another in terms of the kinds, we particularly propose the idea of minimal adequate explanations.

In the priority experience replay algorithm, in view of the uneven quality of randomly sampled experience, resulting in low training efficiency and slow algorithm convergence speed, the joint value function in the value function decomposition algorithm is used to calculate the error, and combined with the number of extractions to calculate priority of samples to speed up algorithm convergence.

### 3.2. Multiagent Sensors Reinforcement Learning Models

**Figure 1:**
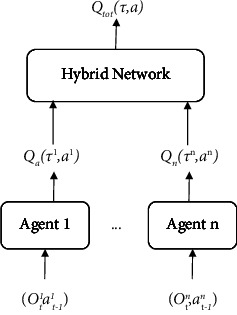
Multiagent reinforcement learning models.

**Figure 2:**
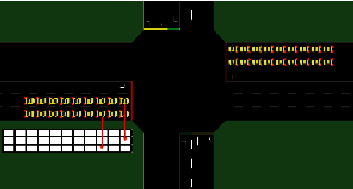
Schematic diagram of vehicle location matrix.

The multiagent sensor reinforcement learning model based on value function decomposition is shown in Figure 1. Based on the value-decomposition networks (VDN) algorithm [19], the original linear mapping is replaced by a nonlinear mapping, and the super network is introduced to add additional global state information to the mapping process to improve the performance of the algorithm [20]. Using the current observation state of each agent which are the intelligent sensors that performs the action of the previous time step as input, a global action-value function is learned through the hybrid network by these intelligent agents, where *τ* is the global state, *α* is the global action. In the multi-intersection variable steering lane scenario, the involved elements such as state space, action space, and reward function are defined. In order to make the input state more realistic and richer, the queue length of the lanes in each direction is adopted as the average waiting time of vehicles, and the ratio of average delay time as indicators. In addition, in order to accurately describe the position distribution of vehicles, the variable steering lane area is discretized and encoded to obtain the vehicle mapping matrix, as shown in Figure 2. The lanes are divided into the same size the grid covers the entire road section. Each grid in grid represents the present state of a vehicle. A value of 1 means that the vehicle exists at the grid, and 0 means that the vehicle does not exists compared with the intersection image directly information as input, this method can compress the data dimension and remove redundant information, thereby speeding up the training practice speed.

In the multi-intersection scenario, the state space expression is defined as follows:

(3) $$\begin{array}{c} S\left(T\right)=\left(L_{L},W_{L},D_{L},L_{s},W_{S},D_{S},M\right)\text{,} \end{array}$$

where *T* is the number of signal cycles, *L*_*L*_, *W*_*L*_, *D*_*L*_ are the average queue length, average waiting time, and average delay time of the lane group in the left-turn direction, respectively, *L*_*s*_, *W*_*S*_, *D*_*S*_ is the average queue length, average waiting time, and average delay time of the lane group in the straight direction, and *M* is the vehicle position mapping matrix.

In the variable steering lane scenario, the straight-left variable steering lane is mainly studied and applied, and the right turn direction is not considered, so the action space is left turn or straight.

The global reward function is defined as the weighted sum of the following metrics.

(1) The average queue length *L* of vehicles in all lanes
(2) The average delay time ratio *D* of vehicles in all lanes
The expression of the single lane delay time ratio *D*_*i*_ is as follows:
  (4) $$\begin{array}{c} D_{i}=1-\frac{v_{lane}}{v_{\max}}\text{,} \end{array}$$
where *v*_*lane*_ is the average speed of vehicles on lane *i*, and *v*_max_ is the vehicle speed maximum speed limit for road *i*:
(3) The average waiting time *W* of vehicles in all lanes. When a vehicle starts to stop and wait, that is, when the speed is less than 0.1 m/s, the parking waiting time of this vehicle starts to accumulate;
(4) *N* is the average number of vehicles on all lanes that left the current lane after the previous action;
(5) *V* is the average speed of vehicles in all lanes leaving the current lane after the previous action. The expression of the average speed is given as follows:
  (5) $$\begin{array}{c} V=\frac{1}{N}\overset{N}{\underset{j=1}{\sum }}v_{j}\text{,} \end{array}$$
where *v*_*j*_ is the average speed of each vehicle.

Assign corresponding weights to the above-mentioned different traffic indicators, and finally calculate the global reward:

(6) $$\begin{array}{c} R=k_{1}L+k_{2}D+k_{3}W+k_{4}N+k_{5}V\text{.} \end{array}$$

In the formula, *k*1, *k*2, *k*3, *k*4, *k*5 are the weight parameters, and the effect of the final traffic condition optimization is set by analyzing the global reward results in 3200 experiments.

### 3.3. Global Reward Decomposition Algorithm

**Figure 3:**
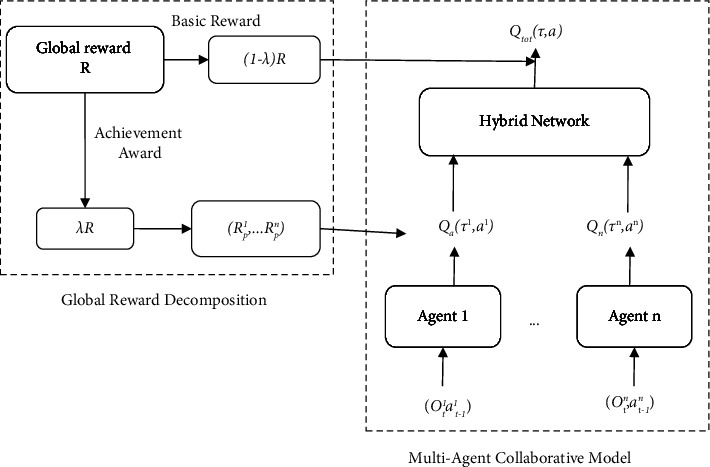
The global reward decomposition algorithm.

The global reward *R* is decomposed into two parts, the basic reward *R*_*b*_ and the performance reward *R*_*p*_ based on the proportion, as shown in Figure 3 for the global reward decomposition algorithm structure. The performance reward is an additional reward that is used to distribute to the agents with larger contributions.

The global reward decomposition function is given as follows:

(7) $$\begin{array}{c} R=R_{b}+R_{p,}\\ R_{b}=\left(1-\lambda \right)R,R_{p}=\lambda R;\;\lambda \in \left[0,1.0\right]\text{.} \end{array}$$

The traditional hybrid network method is used to distribute the basic rewards to each agent which are the intelligent sensors. The performance reward is used to motivate the agents that contribute more in the process of regional cooperative control. The expression of the performance reward obtained by each agent at the current time is given as follows:

Real-world situations like strategic conflict resolution, coordination between autonomous vehicles, and agent cooperation in defensive escort squads all include cooperative multiagent challenges. Such issues may be modeled as dual-interest situations, in which each agent simultaneously seeks to maximize its individual payout (local reward) and the team's performance as a whole (global reward). Two different forms of modern, state-of-the-art MARL algorithms exist. While algorithms like MADDPG and M3DDPG concentrate on optimizing local rewards without any explicit idea of coordination, algorithms like COMA and QMIX strive to maximize the global reward for the success of the group. We first define multiagent cooperation as a joint optimization on reward assignment and demonstrate that each agent has an approximately optimum strategy that decomposes into two parts: one that just depends on the agent's own state and the other that is connected to the states of adjacent agents. CollaQ decomposes each agent's Q-function into a self-term and an interacting term, using a multiagent reward attribution (MARA) loss to regularize training. CollaQ is tested on multiple StarCraft maps and surpasses existing state-of-the-art approaches (such as QMIX, QTRAN, and VDN) by increasing the win rate by 40% while using the same amount of samples.

(8) $$\begin{array}{c} R_{p}^{i}=\left\{\begin{array}{cc} \frac{1}{2L_{T}}\left(L_{S^{\prime }}+L_{L^{\prime }}\right)R_{p}\text{,} & L_{S^{\prime }}<L_{T},L_{L^{\prime }}<L_{T}\text{,}\\ R_{p}\text{,} & other\text{,} \end{array}\right.\\ L_{T}=\frac{V_{out}}{V_{\max}}\text{,}\\ L^{\prime }=\frac{L}{L}\text{.} \end{array}$$

In the formula, *R*_*p*_^*i*^ is the performance reward obtained by the *i*^*th*^ agent, *L*′ is the ratio of the average queuing length of the lane group in a certain direction of the agent to the overall length of the lane, $\bar{L}$ is the average queuing length of the lane group during the execution of the previous decision, *L*_*S*′_ is the overall length of the lane, and *L*_*L*′_ is the ratio of the average queuing length of the straight lane group to the overall length of the lane, the ratio of the average queuing length of the left-turn lane group, *L*_*T*_ the threshold of the determination level of the lane, *V*_*out*_ the maximum traffic flow that can be driven out during the green light of the lane, and the capacity of the lane. *V*_max_ is the maximum traffic flow [21].

All agents are graded when the performance reward is allocated *R*_*p*_, and the performance reward corresponding to each level is different [22, 23]. As shown in formula (8), when the *L*_*L*′_ left turn and *L*_*S*′_ straight lane group queue length ratio and are *L*_*T*_ less than the threshold when the traffic flow of the road section is determined to be small, *L*_*L*′_ and *L*_*s*′_ the performance reward is allocated by the ratio of the average and the larger, more performance rewards should be allocated [24].

### 3.4. Priority Experience Replay Algorithm

Deep learning uses target networks to increase the training's stability. The main training network and the target network are the two networks that the DQN method trains. The difference between the two networks, squared, is the loss the algorithm trains on (often replaced with Huber loss nowadays). The primary training network periodically replaces the target network's weights as training progresses. The target network predicts the optimum Q value from all actions that may be done from the next state in each data sample. This is the desired Q value. To train the *Q* network, the loss is computed using the predicted Q value, target Q value, and observed reward from the data sample. In single-agent reinforcement learning, in order to solve the problem of uneven quality of training samples extracted during the training process, a priority experience playback algorithm [25] was proposed, and the temporal-difference (TD) method was used to measure the importance of samples. The samples with larger errors are set as high priority, and the samples with high priority are extracted for training to improve learning efficiency. In the multiagent where agents are the intelligent sensors the reinforcement learning based on the value function decomposition algorithm, the joint value function can be used to calculate the TD error, and then use it for the calculation of the priority. In order to realize the priority experience playback algorithm, the target network loss *L*_*n*_ must be calculated:

(9) $$\begin{array}{c} {L_{n}=\left(y^{tot}-Q_{tot}\left(S,A\right)\right)}^{2}\text{,}\\ y^{tot}=R+\gamma Q_{tot}\left(S^{\prime },A^{\prime }\right)\text{.} \end{array}$$

In the formula, *S*, *A* are the joint state and joint action of multiagent, *γ* is the attenuation coefficient, *S*′, *A*′ is the joint state and joint action of multiagent at the next moment. The larger the value, *L*_*n*_ the higher the corresponding experience sequence will be priority.

Using *L*_*n*_ as the only indicator to measure the importance of samples may cause some samples to be drawn less frequently due to their small size. Therefore, this study combines the target network loss and the number of times to be drawn *N*_*sam*_ as an indicator to measure the importance of samples. At the same time, considering different empirical the loss value of the target network has a large difference *f*_*Top*_(*L*_*n*_), and it is converted into a dimensionless sorting amount, which is the position of the loss in the increasing sorting. The expression of the final priority PR_*i*_ is given as follows:

(10) $$\begin{array}{c} {\text{PR}}_{i}=\frac{p_{i}}{\sum_{j=1}^{M}p_{j}+\epsilon p_{i}}\\ =f_{Top}\left(f_{Top}\left(L_{ni}\right)+f_{Bot}\left(N_{sam}^{i}\right)\right)\text{.} \end{array}$$

In the formula, *f*_*Bot*_(*N*_*sam*_^*i*^) is the position of the number of extractions in descending order; *ε* ∈ (0,1.0) is the offset of the probability, which is used to correct the situation that the priority is too small to cause the probability of sample selection to be too low.

## 4. Experimental Results and Analysis

In order to verify the effectiveness of the cooperative control algorithm that employs intelligent sensors to alleviate the traffic congestion at multiple intersections methods for efficient quality of service in the next generation IoT applications using reinforcement learning. In the multi-intersection variable steerable lane scenario, the cooperative control BASE algorithm is combined with fixed-time control (FT) and traditional adaptive control algorithm for multi-intersection (MTAC), single-agent reinforcement learning adaptive algorithm (DQN), multiagent reinforcement learning adaptive algorithm (QMIX) and other methods, and analyze the performance of each algorithm in the data set, including algorithm-level reward value, traffic average queue length, average delay-to-time ratio, average waiting time, and average travel time metrics at the level. In cooperative multiagent systems, agents work together to complete tasks in exchange for a group reward as opposed to individual benefits. Credit assignment techniques are often used to differentiate the contributions of various agents in the absence of individual reward signals in order to promote successful cooperation. As credit assignment has recently been widely implemented using the value decomposition paradigm, QMIX has emerged as the leading technology. Robot swarms, autonomous vehicles, sensor networks, and cooperative multiagent reinforcement learning are just a few areas where this technique has found widespread use. In these activities, each agent must learn a decentralized policy through a shared team reward signal because individual incentives are not available. In order to achieve successful collaboration, agents must allocate credit in a discriminatory manner. Credit assignment techniques using cooperative multiagent reinforcement learning have made significant strides in recent years. Value-based approaches have among them demonstrated cutting-edge performance on difficult challenges. Value factorization, which is based on the centralized training with decentralized execution (CTDE) paradigm, has recently gained a lot of popularity. It specifically integrates separate value functions *Q*_*i*_ during centralized training to factorize the combined value function Q_tot_. Decentralized policies may be easily determined during execution by greedily picking individual actions from the local value function *Q*_*i*_. Because *Q*_*i*_ is learned by maximizing the overall temporal-difference error on a single global reward signal, an implicit multiagent credit assignment is accomplished.

### 4.1. Experimental Setup

**Figure 4:**
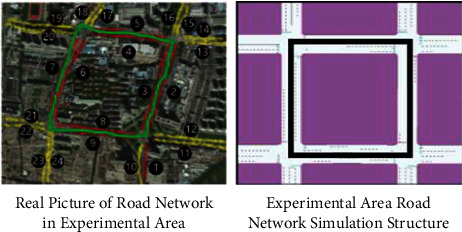
Structure diagram of the experimental road network area.

The experimental equipment configuration of this research is as follows: the CPU is AMD 2.10 GHz, the memory is 16 GB, and the operating system is Windows 10 (64 bit). Simulation experiments are carried out based on the microscopic traffic simulation platform SUMO v1.7.0. Interface to interact with the simulation environment, obtain the traffic status in real-time and adaptively adjust the control strategy of the variable steering lane. As shown in Figure 4, the experimental environment includes 4 intersections and a total of 24 road sections, and the road sections are numbered 1∼9. There are 9 variable steering lanes in total. The section with variable steering lanes consists of 5 lanes: fixed left turn, variable left turn/straight, straight, straight, and right turn lanes. Section number 10∼24 is a conventional road section, there are 15 in total, and the road section adopts a conventional fixed three-lane configuration. The signal cycle of each intersection is the same.

The experimental data set is collected from different traffic capture data of streets, roads, etc. such as upstream and downstream road section codes, capture time, lane number, and license plate number.

In this experiment, traffic flow data were collected at intersections in the urban area. The types of vehicles mainly included cars, station wagons, buses, and large passenger cars. The actual collected vehicle types and numbers were input into the simulation system, which were 24,17,592 cars, 156,008 station wagons, 91,089 buses, and 25,113 large passenger cars, accounting for 89.88%, 5.80%, 3.39%, and 0.93% of the total flow, respectively. In the calculation process of the simulation system, in order to standardize the vehicle position information at the intersection is converted into a discretized encoded vehicle position matrix, which is used as the quantitative input of the reinforcement learning model. In addition, the actual input vehicle type and quantity are converted into standard cars based on the conversion standard. The equivalent conversion coefficients of the models corresponding to standard cars are 1.2 for station wagons, 2.0 for buses, and large passenger cars. As is well known, traffic noise conversion and calculation used to be done using the prediction model in “Specifications for the Environmental Impact Assessment of Highways,” whereas we now know that its levels or grades, as well as its equivalent conversion, can be calculated in a variety of ways using the equivalent conversion coefficient. The findings of the speed survey and their analysis allow us to conclude that the speed computation for all types of vehicles must follow the Gaussian distribution in free traffic flow, allowing us to suggest a speed discretization approach. The approach described above assists in converting various vehicles with varying speeds into car comparable numbers, while overall traffic volume may be converted into that of passenger cars by evaluating themselves at the same noise levels.

In order to ensure the fairness of the comparison algorithm, the network structure and hyperparameter settings of the reinforcement learning algorithm are the same. The value of the discount factor is 0.95, the value of the learning rate is 0.001, the value of the greedy strategy *ε* is 0.05, and the size of the memory bank is 1000. The number of samples for each update is 32, and the model update step is 5 signal cycles. In order to improve the stability of the algorithm, the target network is updated with a delay. The weight replacement step of the target network is set to 30 signal cycles and RMS prop (root mean square prop) algorithm is the update algorithm.

In this study, the weight of global reward decomposition is set to *λ* = 0.4 in the BASE algorithm. Queuing length is used as an indicator to measure the efficiency of road traffic diversion under unbalanced traffic flow, and 32 combinations of weight values are set. In the process of training and testing, the accumulated vehicle queue length is calculated, 100 experiments are carried out for each scheme, and the average queue length is obtained by taking the average of the results. The smaller the average queue length the better the traffic diversion effect a total of 3,200 experiments were conducted using the numerous sensors. The results determine the weight of each influencing factor in the global reward function set in this experiment *k*1, *k*2, *k*3, *k*4, *k*5 are set to −1.0, −0.5, −0.5, 0.5, 1.0, respectively.

### 4.2. Experiment Analysis

**Figure 5:**
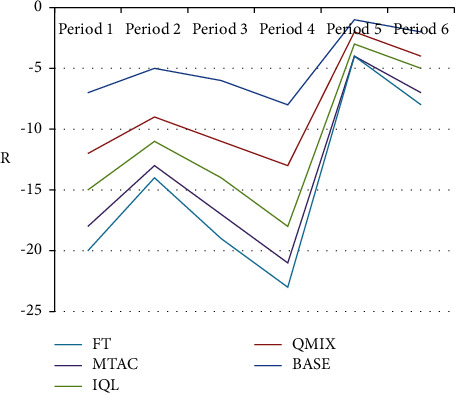
Comparison results of algorithm reward indicators in the test set.

**Figure 6:**
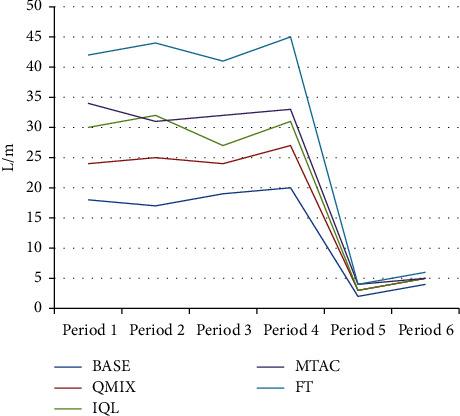
Average queue length.

Performance the test set is data of 6 periods, namely morning peak (periods 1, 2), evening peak (periods 3, 4), and flat peak (periods 5, 6). The performance of each control method is shown in Figures 5 and 6. In Figure 5, R is the reward index. In the data sets of multiple periods, the BASE algorithm performs better overall, followed by the QMIX algorithm, indicating that the multiagent collaborative algorithm is still effective in real scenarios. The performance of BASE, QMIX, IQL, and MTAC is significantly better than that of the timing control scheme (FT), indicating that the adaptive algorithm can effectively adapt to the traffic flow changes in the real scene, and can make appropriate decisions according to the real-time traffic state.

Figure 5 and Table 1 show that the performance of the BASE algorithm in the datasets of multiple time periods is better than that of other algorithms, and the BASE algorithm has a significant lead in the morning and evening peak hours compared to the flat peak hours.

**Figure 7:**
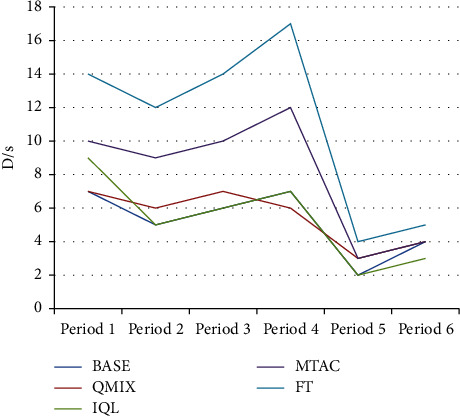
Average delay time.

**Figure 8:**
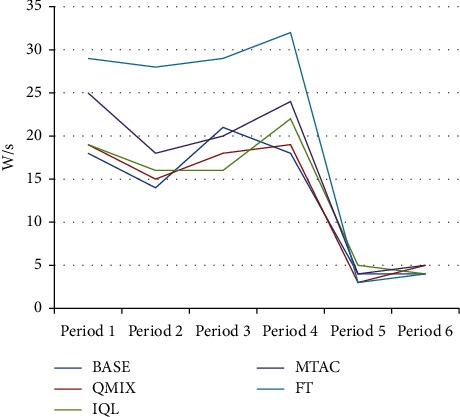
Average waiting time.

As shown in Figure 6 and Table 2, compared with other algorithms, the average queuing length index of the BASE algorithm in the morning and evening peak hours is reduced by 25.76% ∼ 54.97%, and the index in the off-peak hours is reduced by 49.00% ∼ 70.67%, indicating that the BASE algorithm is in the congestion scene. Compared with other algorithms, the average delay time of the BASE algorithm is reduced by 15.54%∼55.09% as shown in Figure 7 and Table 3. In the test sets of 5 and 6 during the peak period, the road traffic in the network is small, the demand for the function of the variable steering lane is weak, and the performance of each algorithm is close. The performance of the algorithm (BASE) in this study still maintains a slight lead. As shown in Figure 8 and Table 4, compared with other algorithms, in the BASE algorithm the average waiting time is reduced by 9.28%∼42.39%, and in peak hours such as time period 3, the traffic state is more congested. The average waiting time of the IQL algorithm on this dataset is slightly better than that of the QMIX algorithm.

**Figure 9:**
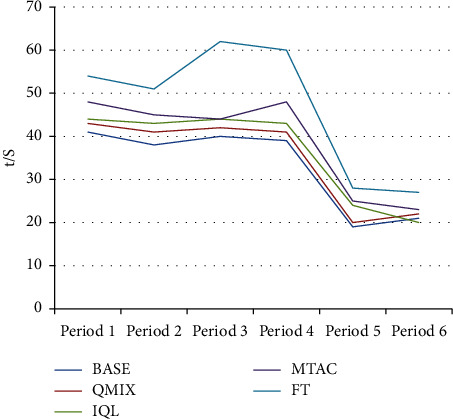
Average travel time.

It can still maintain the leading performance, which further proves the effectiveness of the improved algorithm. As shown in Figure 9 and Table 5, the average travel time is reduced by 6.44%∼29.93% compared with other algorithms, and the performance is stable under the test set of 6 periods, always has better performance. Comparison results of each traffic index in the test set are shown in Figures 6to 9.

**Figure 10:**
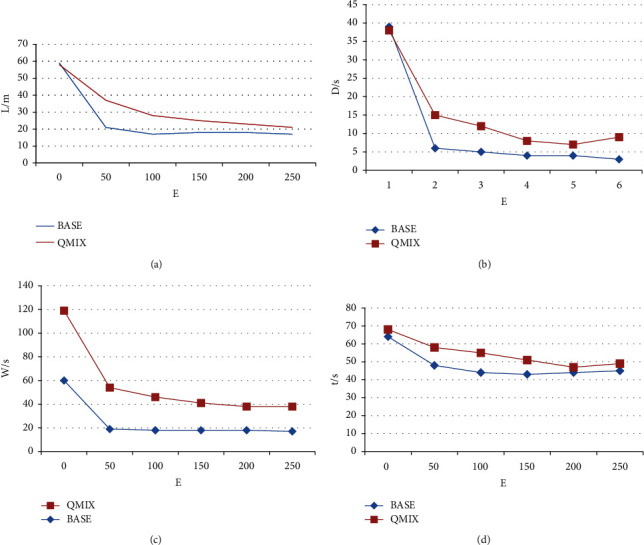
(a): Average queue length. (b): Average delay time. (c): Average waiting time. (d): Average travel time.

The best performance of the algorithm in this study verifies the improvement of the multiagent collaborative algorithm for the multi-intersection variable-guidance lane scene: global reward decomposition, the proposed algorithm in this study can learn the policy better than the QMIX algorithm performance of the training process by comparing the BASE algorithm with the QMIX algorithm, the performance indicators of the training process are tested. The comparison of the average accumulated reward value index is shown in Figure – and Table 6. In Figure 10, E is the number of iterations.

The changes in traffic indicators during the training process are shown in Figure 8. Traffic indicators such as average queue length, average delay time, average waiting time, and average travel time change with the training process of the algorithm model. The indicators are significantly reduced, the traffic state is gradually optimized, and the two multiagent collaborative algorithms can converge. At the same time, according to the downward trend of each traffic indicator in the training process and the final convergence state, it can be found that the global reward decomposition and priority experience playback are applied. The BASE algorithm of algorithm has a faster convergence effect and better optimization performance, which can effectively adapt to the traffic flow of multi-intersection scenarios, and realize traffic state optimization of the local road network.

## 5. Conclusion

Computational intelligence is built on several learning and optimization techniques. Therefore, an emerging trend of major significance in the future generation of IoT applications is the integration of new learning methods, such as opposition-based learning, optimization approaches, and reinforcement learning. This research proposes a multiagent reinforcement learning-based collaborative control method employing intelligent sensors for variable-guidance lanes at multiple intersections. This method improves the performance in congested scenarios through a global reward decomposition algorithm and improves learning efficiency through a priority experience replay algorithm. Cooperative control of variable-guidance lanes at multiple intersections: compared with other control methods, this method has better effects in reducing the average queue length, average delay time, average travel time, etc., while convergent faster, and therefore, enhancing the quality of service in the next generation of IoT applications.

The follow-up work includes combining the algorithm with traffic signal control and performing joint optimization in the two dimensions of time and space to further improve the traffic capacity of multi-intersection scenarios.

## Figures and Tables

**Table 1:** Comparison results of algorithm reward indicators in the test set.

BASE	QMIX	IQL	MTAC	FT
−7	−5	−3	−3	−2
−5	−4	−2	−2	−1
−6	−5	−3	−3	−2
−8	−5	−5	−3	−2
−1	−1	−1	−1	0
−2	−2	−1	−2	−1

**Table 2:** Average queue length.

BASE	QMIX	IQL	MTAC	FT
18	24	30	34	42
17	25	32	31	44
19	24	27	32	41
20	27	31	33	45
2	3	3	4	4
4	5	5	5	6

**Table 3:** Average delay time.

BASE	QMIX	IQL	MTAC	FT
7	7	9	10	14
5	6	5	9	12
6	7	6	10	14
7	6	7	12	17
2	3	2	3	4

**Table 4:** Average waiting time.

BASE	QMIX	IQL	MTAC	FT
18	19	19	25	29
14	15	16	18	28
21	18	16	20	29
18	19	22	24	32
4	3	5	4	3

**Table 5:** Average travel time.

BASE	QMIX	IQL	MTAC	FT
41	43	44	48	54
38	41	43	45	51
40	42	44	44	62
39	41	43	48	60
19	20	24	25	28

**Table 6:** Average accumulated reward value index.

Average queue length		Average delay time		Average waiting time		Average travel time	
BASE	QMIX	BASE	QMIX	BASE	QMIX	BASE	QMIX
59	58	39	38	60	59	64	68
21	37	6	15	19	35	48	58
17	28	5	12	18	28	44	55
18	25	4	8	18	23	43	51
18	23	4	7	18	20	44	47
17	21	3	9	17	21	45	49

## Data Availability

The data shall be made available on request.
